# Book Reviews

**Published:** 1833-02

**Authors:** 


### CRITICAL ANALYSES.

**Quæ laudanda forent, et quæ culpanda, vicissim,

Illa prius, cretâ; mox hæc, carbone, *notamus*.—Persius.**

*A Treatise on the Urethra; its Diseases, especially Stricture, and their Cure.* By Benjamin Phillips, author of "A Series of Experiments made to demonstrate that Arteries may be obliterated without Ligature, Compression, or the Knife."—8vo. pp. 319; two plates. Longman and Co., London, 1832.

The subject selected by Mr. Phillips is one devoid neither of interest nor importance; and the manner in which it has been treated in his essay is plain, practical, and useful. We are not amongst those carping critics who are continually craving for something new; whose morbid appetites refuse to digest wholesome and nutritious food, merely because to some parts are wanting the excitement of novelty; for we are well aware that value often holds an inverse ratio to newness. But, even with all our tolerance in these respects, we think we could have excused the (shall we say, vain) repetition of much of the anatomical details; and the proportion of quoted pathological matter we think rather more than the little spice the author has mixed with it required: for the surgical epicures, the menstruum is too abundant; and for the Podalirian babes, the pure milk of science flows in much too scanty streams. Yet what could the author do? We sympathize with him in his dilemma; for, had these materials been rejected, to what a ghost-like *libellum* this portly volume would have shrunk!

We are glad to find, by some incidental notices in the earlier pages, that the doctrine of *epigenesis* is making way, and becoming familiar in this country, where, but a short time since, the very meaning of the word, as anatomically applied, was in general but little understood. By means of this doctrine, the structure of the prostate is here very prettily explained.

"Before I terminate this chapter, I may be permitted to say a few words on the development of the prostate, as established by Serres; because it may serve to throw some light on a subject which has given rise to much discussion: I mean the existence or non-existence of a third lobe, so strongly insisted on by Sir E. Home, and previously noticed by Morgagni. Though the prostate does not actually constitute an essential part of the urethra, yet its connexion with it is so intimate, and the question appears of so much importance, that I shall briefly describe the phenomena of the development of that organ.

"Primitively, in the human embryo, we do not find the prostate; neither do we perceive it until the termination of the second month. At that period it is formed of four lobes; and this multilobular division corresponds to that of other organs in the embryo, as the kidneys, &c. From the fourth to the fifth month, the two posterior lobes unite to form one, the prostate at this period appearing to consist of three lobes; and from the sixth to the eighth month, all these lobes unite, and form, as in the kidney, a unique organ, which embraces the origin or part of the origin of the urethra; yet, by an attentive dissection, we may discover, as in the kidney, traces of the primitive organization. It is evident, then, that if the union of these three portions be, under any circumstances, arrested, there may be presented a posterior and middle portion, which may be termed a middle lobe.

"When suppuration attacks those organs which originally present a multilobular character, and which afterwards, during the progress of organization, lose that character, and assume the appearance of unique organs, it is found that its ravages are at first directed to those points which were last formed; and, when their destruction is effected, the organ again presents its multilobular character. This often occurs in the kidney; and it is to a similar phenomenon that I refer the occasional appearance of the 'third lobe' of the prostate, which is very rarely demonstrable, except during a pathological condition of the organ." (P. 21.)

The disputed point as to the reproduction of mucous membranes, is very fairly and fully discussed by our author, and answered in the affirmative; an affirmation he thinks a necessary precursor to his advocacy of the caustic system of treating strictures.

The most prominent features of the third chapter are, the substitution of the term URETHRITIS for *gonorrhœa*, and the adoption of ORCHITIS for *hernia humoralis;* a change of which our author strongly urges the propriety, and to which we see no other objection than the certainty that they will not be generally employed. Our author is right, and on this point custom is wrong; but then custom is stronger than our author. However, he shall have the full advantage of his argument.

"In order to avoid misconception, I think it necessary to state that I intend to employ the word Urethritis for the purpose of designating all those affections of the urethra accompanied by a mucous, muco-purulent, or purulent, discharge, and presumed to be dependent on inflammation of the mucous membrane by which that canal is invested.

"It is not from a simple desire for innovation that I propose this change in the nomenclature of these diseases: for no one can be more entirely sensible of the inconvenience which is occasioned by such changes than I am: but, when I reflect upon the inapplicability of the terms now employed to convey to the mind a knowledge of those particular diseases to which they are applied, I cannot help feeling convinced of the propriety of adopting some term which would be more precise in its signification than those now used. When I look at the terms gonorrhœa, blennorrhagia, and blennorrhœa, I not only see that, as terms, they are radically incorrect, even when used for the purpose of distinguishing any particular period of the disease, but, when applied, as they ordinarily are, to the disease generally, without reference to particular periods of its duration, I can no longer hesitate in adopting a term which is undeniably more precise in its signification and better adapted to the object required. To specify particular forms, I shall use the prefix acute or chronic, contagious, or non-contagious." (P. 63.)

The section on the causes of urethritis is so well written that we cannot avoid quoting it entire.

"Urethritis is produced by different causes. The first of those to which I shall allude is purely mechanical, the second chemical, and the third virulent, by which I mean a disease produced by the application, upon the mucous membrane of the urethra, of contagious matter, either by connexion with a person suffering from contagious urethritis, or by any other mode of contact.

"Among the mechanical causes are venereal excesses between two individuals in whom the genitals are perfectly healthy. This may produce, in one or both, a more or less intense urethritis. This is a fact which constant observation verifies. We have seen a woman, in the apparent enjoyment of the highest health, a connexion with whom produced urethritis in all who had connexion with her, and who yet never suffered from urethral or vaginal disease. Cullerier mentions the case of a girl who had never been affected with urethritis or vaginitis, and in whom the organs, when examined by him, were perfectly healthy, who communicated to a young man urethritis of an extremely acute character; and examples of this kind might easily be multiplied, were it necessary. Masturbation, if violent, will produce urethritis; and indeed the disease is not unfrequent in girls addicted to this vice. In boys the disease is less frequently produced by this vice, from obvious causes; for it is produced only by the irritation occasioned by violent or continued friction of the mucous surface in girls; and, as contact with the interior of the urethra is not produced in the male, the difference is easily understood. The violence suffered by young girls from libertines very frequently produces a similar affection of the vagina, while the authors of such violence may be exempt from the disease, the contusion and distention of the sexual organs being sufficient to occasion the discharge. This is a fact which should be borne in mind by medical practitioners who are required to give evidence in courts of justice, in cases where forcible attacks have been made upon females. Great circumspection should be used in asserting the existence of contagion; for the person accused may prove the absence of the disease in his own person, and may exculpate himself by the testimony of his accusers. The accused may answer thus, 'You say this infant has a contagious discharge, and that the contagion has been communicated by me; but I am not suffering from the disease, and it is therefore impossible that I can have committed the violence imputed to me.' This has occasionally occurred. Two summers ago, an individual was accused, in Paris, of having committed violence on a young child, and of having communicated to her a contagious affection: he was examined by order of the court, and found perfectly healthy. It is true that cases frequently occur where violence is committed on young children by persons who are suffering from contagious urethritis, and who are in some instances prompted to the commission of the crime by a belief, still common among the vulgar, that by communicating the disease to a virgin they remove it from themselves.

"We may also enumerate, among the mechanical causes of urethritis, contusions of the perineum, the presence of stone in the bladder or in the urethra, strictures, and, in fact, every thing which may mechanically irritate the urethra, or the parts to which it is related, either directly or sympathetically. Urethritis produced from these causes is, however, rarely very acute (though much more frequent than is generally supposed,) and readily ceases when the exciting cause is removed.

"Chemical irritants also excite inflammation of the membrane of this canal, and produce augmented secretion; but urethritis from these causes is so rare, that we have no other well authenticated cases than those produced by Swediaur on his own person. He produced a very intense urethritis, altogether similar in character to that which has a venereal origin, by injecting into the urethra a weak solution of ammonia.

"It is very common to see urethritis succeeding to connexion with a woman during the existence of the menstrual flux; and, perhaps more frequently to connexion with women who have lochial or leucorrhœal affections.

"The cause considered by the world, and even by medical men, as the most common origin of urethritis, is the application of the substance secreted by mucous membranes which have been subjected to the contact of contagious matter: and this is, I apprehend, the only manner by which a contagious urethritis can be produced.

"The internal causes by which urethritis may be produced are not less numerous. Irritations of the alimentary canal, cutting the teeth (this is, however, an unfrequent cause, and is more rare in boys than girls,) certain aliments or medicinal substances, as beer, turpentine, tea, asparagus, cantharides, and spices, may occasion in the urethra an irritation which not only renders the emission of urine painful, but produces an abundant discharge from the urethra. Schenk speaks of a man who could produce a gonorrhœa at will by eating cress.

"'Irritations of the mucous membrane of the air-passages are often accompanied by urethritis.' If it be true that such diseases are occasionally accompanied by affection of the urethra, we may have less difficulty in admitting, with Blassius, that urethritis may be epidemic. I am not satisfied of these complications ever existing; but they have been observed by Fabre, Goulard, Morgagni, and Noel, in some very hot seasons which were very rapidly succeeded by cold, humid weather. We know that these sudden changes almost invariably affect mucous membranes generally; and I confess I can see no sufficient reason why they may not similarly affect that of the urinary passages. It may be said that the latter is less exposed to the action of those changes. That is, no doubt, true; and it is equally true that it is here much more rarely produced: but we see many persons with clothing insufficient to protect this membrane more effectually than that of the mouth.

"Again, diseases of the skin accompany or alternate with urethritis. Of these diseases, lepra appears to be the most frequent concomitant. That urethritis which is said to be produced by, or to accompany, lepra, was particularly frequent in the fifteenth century. May we believe that it is this which is so well described by Moses? Still, in spite of the imposing authorities who advocate such doctrines, in spite of their conviction that the disease produced by such causes cannot be distinguished from those succeeding to contagious intercourse, in defiance of their conviction that the symptoms, the varieties, the complications, the consequences, are identical, that the matter exhaled resembles perfectly in the one case to the other (being dependent in either case on the degree of inflammation,) I must elevate my feeble voice against this doctrine of identity; for, varied as have been my opportunities of watching these diseases, frequent as have been the opportunities afforded me of ascertaining the opinions of the greatest authorities on the subject, I feel no hesitation in laying down a positive opinion, in saying that the discharge proceeding from an urethritis produced by these causes can never produce a contagious disease of a similar character in the person upon whom it has been applied.

"In modern times, up to the present moment, I know only one person who affected to be able to distinguish an urethritis of a contagious character from that which was not so. Wedekind says that he has established a character by which it may be distinguished. The character consists in this, 'that, in men who have been infected during coitus, there is developed, immediately behind the meatus urinarius, two small lenticular tubercles, situated the one by the side of the other, and very sensible to the touch. In examining,' says he, 'many subjects who, after a suspected connexion were apprehensive of having contracted a contagious urethritis, I can each time announce before the appearance of the disease (after the formation of the tubercles,) whether the affection be contagious or non-contagious. When I do not feel these small bodies, contagious urethritis will not supervene, whatever may be the extent of the itching in the gland and the prepuce, and notwithstanding the heat in urining. They enlarge and become more sensible when the lips of the orifice of the canal begin to swell, and a mucous viscid humour bathes the gland. They enlarge at the commencement of dysury and the period of inflammation. These tubercles are always the most sensible part; they diminish in violence and sensibility in proportion as the disease decreases; but we cannot regard it as being entirely dissipated until they have become quite insensible. All appearance of the disease may disappear, not only for some days, but for entire months, the patient may believe himself completely cured; but a cold, an error in regimen, fatigue, excess in coitus with a healthy person, in fact, any thing which may irritate the urethra, may cause a return of the discharge, so long as these two tubercles are more or less sensible.' Such is the opinion of Wedekind. I need hardly say that his opinion is without foundation; for these tubercles are developed every day by simple irritation, and in persons who have never suffered from urethritis.

"The question has long been discussed, whether the matter secreted at the surface of a primitive venereal ulcer ever produces 'gonorrhœa,' and if the matter of 'gonorrhœa' ever gives birth to chancre? and, after all the elaborate investigations which have been resorted to for the purpose of determining this question, it is yet undecided." (P. 64.)

We shall pass by the sections on the symptoms and treatment of Urethritis, with the running observation, that our author speaks strongly, and with justice, of the benefits derived from the use of lunar caustic in protracted urethral discharges: he recommends the application in substance, but we believe the best mode of using this powerful agent, in most cases, will be found to be smearing the bougie with a strong unguent of nitrate of silver, such as is used so successfully in ophthalmia.

From the chapters on the "definition, origin, classification, situation, and symptoms of Stricture," and on the "history of the Treatment of Strictures," with a "review of the various Remedies, dilatation, (bougies, and sounds,)" we shall make but a single extract; and yet that which closes these dissertations will suggest to our well-informed readers the course of the argument as well as much more lengthened extracts.

"I have now passed in review the methods of treating strictures by bougies and by sounds, and have, I submit, established their inapplicability in the vast majority of cases. I have shown that by far the greater number of strictures are an effect of induration existing in the parietes of the canal; that this induration can only be removed by dilating bodies, through the medium of absorption; that, if induration has long existed, absorption can no longer be performed, and that bougies or sounds can therefore succeed in the earliest stages of the disease only; whilst it is notorious that application for medical assistance is rarely made until the disease has acquired a formidable character. Absorption being, then, available for the cure of strictures in a very early stage of the disease only, and compression being, as I apprehend, wholly inefficient for the removal of the obstruction, there remains but one mode by which, in the case of organic stricture, we can restore the canal to its original capacity, and that mode is destruction of the stricture; and this is effected by means of a caustic substance or a cutting instrument." (P. 192.)

Mr. Phillips has invented an instrument which seems well adapted for the application of caustic to the stricture to be destroyed, without injuring more than possible the sound parts adjacent. This consists essentially of a canula, in which is concealed a rod bearing at its end the caustic, which can be protruded at pleasure, and is thus much more manageable than the common armed bougie. With regard to the subsequent treatment, he observes,

"I am decidedly of opinion, that when stricture has been completely destroyed, either by caustic or incision, the use of bougies has ordinarily a tendency to retard the completion of the cure; and we always find that patients do not urine without inconvenience until the use of bougies has been entirely abandoned. I would here, then, distinctly place as a principle (to which I conceive observation and reasoning tend,) that dilatation is generally useless as an auxiliary means to cauterization, and may sometimes be injurious." (P. 213.)

On the subject of Caustic, he continues, with reference more especially to another new instrument,

"As the great majority of strictures are the effects of induration of the tissues, which has generally existed for a considerable time before application is made to the practitioner, who is seldom indeed consulted until the stream of urine is so inconsiderable as to occasion much inconvenience to the patient, and as it has been shown that dilatation has then ceased to be effective for the removal of the disease, either caustic or cutting must be resorted to. Much of the obloquy that has been heaped upon cauterization has unquestionably been occasioned less by the action of the caustic than by the unskilful mode in which it has been applied; and it is certain that, from the improvement in the instruments by which the application is made, no apprehension need, in the present day, be entertained that the caustic will come in contact with the healthy tissue. I have always felt the strong objections which present themselves to the application of caustic to the anterior surface of a stricture, as well as the very serious accidents to which such application may give rise. It has, therefore, been for some time an object of anxiety with me to render the treatment by caustic free from these objections, by devising some means of penetrating the stricture, however small may be its orifice, and effecting its destruction from within outward; and now I submit the following modifications of treatment in such cases.

"Should the orifice of the stricture be too small to admit of the introduction into it of an instrument bearing the caustic, it may be immediately enlarged sufficiently, by the introduction of a cutting instrument, which will be found described and represented at the end of the work. Immediately after withdrawing the instrument, which will sufficiently enlarge the orifice, the instrument bearing the caustic should be introduced, and the application made to the interior of the stricture. With these modifications, a case of stricture from advanced induration can scarcely, I think, occur, in which the use of caustic may not be resorted to with the most perfect safety and the most certain success. Even when the orifice of the stricture is so small as to admit of the evacuation of urine only drop by drop, it may be instantly enlarged, the caustic applied, and all apprehension of retention immediately removed. Taking, then, into consideration all the circumstances connected with cauterization, firmly impressed as I am of the necessity of entirely destroying the induration, convinced too, from experience, of the perfect safety with which caustic may be applied, and the facility with which, under the modification I propose, it may be placed in contact with the interior of every stricture, feeling that this is the desideratum which has been long sought, and that nothing now appears wanting to ensure the success of the application, I earnestly recommend its adoption by the profession, asking from the operator nothing but the precautions which I have recommended. I here terminate the remarks I have to make on cauterization, and shall proceed to describe the treatment by incision." (P. 216.)

The instrument here referred to is called a *urethrotome*, and consists of a circular knife, enclosed in a canula, and guarded by a probe-pointed stiletto, which, if possible, should be passed through the stricture: but, where this can be done, it must be a question whether caustic should not supersede its use. The mode of operating with it, and the cases for which it is destined, are described as follows:

"In those cases where the stricture is of a valvular kind, where it retreats before the bougie, and where, from this circumstance and its trifling extent, some uncertainty would attend any attempt to cauterize it, the cutting instrument I have invented may be employed with the most complete success. Where the induration has become so excessive that the indurated matter acquires almost a horny texture, and where the extent of surface which it occupies is inconsiderable, and, lastly, when any circumstance renders it necessary that the stricture should be very rapidly destroyed, in all these cases I do not hesitate in recommending the employment of this instrument. By the operation which I have introduced, the obstacle is instantly removed, after which an elastic catheter is passed into the bladder, for the purpose of protecting the incised portion of the canal from the irritation which would be produced by the escape of the urine, during the evacuation of the contents of the bladder. The instrument which I have constructed for the purpose is described in the Appendix, where a representation thereof is given. It presents a circular cutting edge, is introduced into the urethra in a canula, and when the canula is in contact with the stricture, a probe, or stilet, situated in the centre of the cutting portion, is gently introduced into the orifice of the stricture, and serves to maintain the instrument in the proper position in the canal. The cutting instrument is then advanced, placed in contact with and pressed against the stricture, a circular motion being at the same time given to it, similar to that given to a trephine, and in two or three moments the stricture is removed and the canal free. The operation may be performed with much facility, is wholly unattended by danger, and the pain is not much more considerable than that which accompanies the application of caustic.

"It possesses then, in the cases I have mentioned, the following advantages over caustic: that of being more completely under the control of the operator, of creating less inconvenience than is occasioned by cauterization (a single operation being, under any circumstances, sufficient for the removal of the disease), and of being applicable in cases when caustic, either from excessive induration, nervous irritability, or other circumstances, cannot be so advantageously applied." (P. 221.)

"There is one more circumstance connected with cauterization and incision, to which I shall allude; and that is the apprehension entertained by many persons of the occurrence of hemorrhage, which they think likely to succeed the judicious application of either of these remedies. No hemorrhage, which is likely to succeed the skilful application of either of these remedies, need occasion any disquietude, except in cases of extreme debility: for, before the blood-vessels enter the mucous system, they become capillary. If, however, hemorrhage should prove troublesome, cold water may be dashed upon the penis and surrounding parts, or injected into the urethra, and one or other of those applications will generally be found effectual to arrest the discharge. If, however, the hemorrhage have proceeded to such an extent as justly to inspire inquietude, if the means I have already recommended should have failed, it may be necessary to resort to pressure; and for this purpose we introduce into the canal of the urethra the largest size bougie which can be admitted; and if this should be insufficient, it should be accompanied by external pressure along the penis and perineum." (P. 223.)

In the classification of strictures there are some interesting generalizations.

"The observations were made in 119 cases of stricture.

"Of these, 117 had suffered from urethral discharges.

In 49, astringent injections had been used.
5 had been subjected to forced catheterism.
29 had, during the progress of the disease, complete retention of urine.
6, retention of spermatic fluid.
6, catarrh of the bladder.
7, paralysis of the bladder.
13, urinary fistula.
6, urinary abscess, with fistulous communication with the urethra.
4, spasmodic affection of the urethra without organic affection.
4, urethral hemorrhage, previous to and during treatment.
14, tumefaction of the prostate.
5, tumefaction of the testicle.
8, diseases of the skin.

"This is then a fair estimate of the frequency with which individual complications occur. Of the 119 cases,
36 were treated by dilatation; of whom
11 only were cured by that means, and, of the remainder,
19 were afterwards cauterized successfully.
81 were cauterized primarily; of these
72 were successfully treated by this means.
Of the 72 who were successfully cauterized,
13 had relapses.
Of the 72 who were cauterized.
65 were treated by consecutive dilatation.
Of these, 13 had relapses.
In 7, consecutive dilatation was not employed, and no relapse occurred.
5 cases were incised by the urethrotome,
4 of them successfully, the obstruction being at once removed: in the other the operation was not completed.

"In seven cases of cauterization, when the cure was permanent, and in the four cases by incision, consecutive dilatation was not employed.
Of the thirty-six cases treated by dilatation,
4 were in persons above 60,
20, in persons between 40 and 60.
8, in persons between 25 and 35,
4, in persons under 25.

"Of those above 60, 2 failed; amelioration only having been produced.

"Of those between 40 and 60, 4 were cured and 16 failed; the reappearance of the disease soon following the suspension of the treatment.

"Of those between 25 and 35, 3 were cured and 5 failed, the disease soon reappearing.

"Of those under 25, all were cured.

"In those nine cases where cauterization failed, the parties were of the following ages: 42, 45, 58, 60, 76, 63, 69, 76, 77.

"The patients of sixty-nine and seventy-seven died of cerebral affection during the treatment; the patient of seventy-six became impatient and was lost sight of; the four younger patients, in consequence of a relapse, refused to undergo further treatment: of the other two I can give no account.

"In the 72 cases where cauterization was successful,

8 were above the age of 60; of these were relapses, 3.
24 were between 45 and 60; of these were relapses, 6.
22 were between 35 and 45; of these were relapses, 3.
18 were between 20 and 30; of these was relapse, 1.

In the five cases of incision,

4 were between 45 and 60; these were all cured.
1 between 40 and 45, not cured.

The cases then, during the progress of which total retention occurs, are in the proportion of one in four. Those in which perineal abscess occurs are as one in six. Those in which fistulæ occur are as one in nine. Those in which spasmodic strictures, in the absence of organic disease, occur, are as one in thirty. Those in which urethral hemorrhage occurs, in a similar proportion. The cures by dilatation are as one in three. The cures by cauterization are as fourteen or fifteen in sixteen. I am not in a condition to state the proportion of relapses after dilatation: those after cauterization are as one in seven or eight; and the age which afforded the greater proportion of relapses was above sixty. Of the cases of stricture which are presented, the proportion occurring at given periods of life is as follows:

From sixty to seventy, one in twelve.
—— fifty to sixty, two in eleven.
—— forty to fifty, three in eleven.
—— thirty to forty, four in thirteen.
—— twenty to thirty, one in seven." (P. 226.)

The chapters on the "Treatment of Retention of Urine," "Puncture of the Bladder and Urethra," "Abscesses, Fistulæ," "Diseases of the Prostate," and the appendix of Cases, although necessary, we suppose, to complete the author's design, do not afford any thing of sufficient interest for translation to our pages. We, however, think, when condemning the operation of opening the urethra on the vesical side of the stricture, the author should have referred to that very successful modification which has been several times performed by Mr. Mayo, and of which we shall hope to have a full account in that gentleman's forthcoming work "on the Diseases of the Pelvic Viscera."

*Medico-Chirurgical Transactions.* Vol. XVII.—8vo. pp. 527; four plates. Longman and Co., London.

(Concluded from page 43.)

THE "*Account of some Experiments on the use of Styptics in Hemorrhage from Arteries,*" by Mr. CÆSAR HAWKINS, will be found replete with interest: they confirm the views we ventured to take when MM. TALRICH and HALMA-GRAND had persuaded themselves, and wished to persuade the world, that they had discovered a potent styptic, which could arrest the most violent arterial hemorrhage. Mr. Hawkins has shown that ordinary styptics, such as nutgalls, nay, even plain cold water, or simple pressure, will effect as much as their boasted "Liquide hæmostatique." We place little reliance on any hæmostatique application, when large arteries are wounded, besides the ligature: nevertheless, we publish, among our Collectanea, some further account of reputed potential styptics.

We would fain enrich our pages with extracts from Mr. WOOD's essay "*on some Effects of Inflammation of the Membranous Lining of the Larynx, with Suggestions relative to the Operation of Bronchotomy, and incidental Remarks on Spasm and Wounds of the Throat;*" as well as from Mr. HEWLETT's "*Case of extensive Ovarian Disease;*" but space forbids: we cannot, however, pass by Mr. BRODIE's "*Cases of Chronic Abscess of the Tibia,*" for they have the recommendation both of importance and originality.

"CASE I. Mr. P., about twenty-four years of age, consulted me in October 1824, under the following circumstances.

"There was a considerable enlargment of the lower extremity of the right tibia, extending to the distance of two or three inches from the ankle-joint. The integuments at this part were tense, and they adhered closely to the surface of the bone.

"The patient complained of a constant pain referred to the enlarged bone and neighbouring parts. The pain was always sufficiently distressing: but he was also liable to more severe paroxysms in which his sufferings were described as most excruciating. These paroxysms recurred at irregular intervals, confining him to his room for many successive days, and being attended with a considerable degree of constitutional disturbance. Mr. P. described the disease as having existed more than twelve years, and as having rendered his life miserable during the whole of that period.

"In the course of this time he had been under the care of various surgeons, and various modes of treatment had been resorted to without any permanent advantage. The remedies which I prescribed for him were equally inefficacious. Finding himself without any prospect of being relieved by other means, he made up his mind to lose the limb by amputation; and Mr. Travers having seen him with me in consultation, and having concurred in the opinion that this was the best course which could be pursued, the operation was performed accordingly.*

"On examining the amputated limb, it was found that a quantity of new bone had been deposited on the surface of the lower extremity of the tibia. This deposition of new bone was manifestly the result of inflammation of the periosteum at some former period. It was not less than one third of an inch in thickness, and when the tibia was divided longitudinally with a saw, the line at which the new and old bone were united with each other was distinctly to be seen.

"The whole of the lower extremity of the tibia was harder and more compact than under ordinary circumstances, in consequence, as it appeared, of some deposit of bone in the cancellous structure, and in its centre, about one third of an inch above the ankle, there was a cavity of the size of an ordinary walnut, filled with a dark-coloured pus. The bone immediately surrounding this cavity was distinguished from that in the neighbourhood by its being of a whiter colour, and of a still harder texture, and the inner surface of the cavity presented an appearance of high vascularity. The ankle-joint was free from disease.

"It is evident that if the exact nature of the disease had been understood, and the bone had been perforated with a trephine, so as to allow the pus collected in its interior to escape, a cure would probably have been effected, without the loss of the limb, and with little or no danger to the patient's life. Such, at least, was the opinion which the circumstances of the case led me to form at the time; and I bore them in my mind, in the expectation that at some future period I might have the opportunity of acting on the knowledge which they afforded me, for the benefit of another patient.

"Case II. Mr. B., at that time twenty-three years of age, consulted me in the beginning of February 1826.

* "It is right that I should state briefly the termination of the case; especially as the circumstances attending it were probably connected with a peculiar condition of the nervous system, occasioned by the long continuance of the local disease. Unfortunately, I preserved no notes of this part of the case at the time, but I have no doubt that my recollection is accurate as to the following particulars. The patient bore the operation with the utmost fortitude, but immediately afterwards he was observed to become exceedingly irritable, restless, and too much disposed to talk. Unfortunately, in the evening there was hemorrhage from the stump; which ceased, however, on the removal of the dressings and coagulum. During the night he had no sleep; and on the following day he was restless and incessantly talking, with a rapid pulse. These symptoms became aggravated. There was no disposition to sleep, and the pulse became so rapid that it could be scarcely reckoned. Until the third or fourth day, the tongue remained clean and moist; after this period it became dry and somewhat brown, and there was constant delirium. The pupils were widely dilated, and the sensibility of the retina was totally destroyed, the glare of a candle not being perceptible even when held close to the eye. Death took place on the fifth day after the operation. No morbid appearances were observed on the post-mortem examination.

"There was a considerable enlargement of the right tibia, beginning immediately below the knee, and extending downwards so as to occupy about one third of the length of the bone.

"Mr. B. complained of excessive pain, which disturbed his rest at night, and some parts of the swelling were tender to the touch. The knee itself was not swollen, and its motions were perfect.

"He said that the disease had begun more than ten years ago, with a slight enlargement and pain in the upper extremity of the tibia: and that these symptoms had gradually increased up to the time of my being consulted. Various remedies had been employed, from which, however, he had derived little or no advantage.

"Having inquired into the circumstances of the case, I was led to regard it as one of chronic periostitis; and I adopted the following method of treatment: An incision was made longitudinally on the anterior and inner part of the tibia, extending from the knee four inches downwards, and penetrating through the periosteum into the substance of the bone. The periosteum was found considerably thickened, and the new bone, which had been deposited beneath, was soft and vascular. The immediate effect of the operation was to relieve the pain which the patient suffered, so that he slept well on the next and every succeeding night. After this I prescribed for him a strong decoction of sarsaparilla. The wound gradually healed, and it was for some time supposed that a perfect cure had been accomplished.

"The enlargement of the upper extremity of the tibia, however, never entirely subsided; and in August 1827, pain was again experienced in it. At first the pain was trifling, but it gradually increased, and when I was again consulted, in January 1828, Mr. B. was unable to walk about, and quite unfit for his usual occupations. At this period the pain was constant, but more severe at one time than at another, often preventing sleep during several successive nights. The enlargement of the tibia was as great as when I was first consulted; and the skin covering it was tense and adhering more closely than is natural to the surface of the bone.

"Some remedies which I prescribed were productive of no benefit. The patient's sufferings were excruciating, and it was necessary that he should, if possible, obtain immediate relief. The resemblance between the symptoms of this case and those of the case already described were too obvious to be overlooked. It appeared highly probable that they depended on the same cause; and I therefore proposed that the bone should be perforated with a trephine, in the expectation that an abscess would be discovered in its interior. To this the patient readily assented, and accordingly the operation was performed in the beginning of March 1828.

"My attention was directed to a spot about two inches below the knee, to which the pain was particularly referred. his part of the tibia was exposed by a crucial incision of the integuments. The periosteum now was not in the same state as at the time of the former operation. It was scarcely thicker than natural, and the bone beneath was hard and compact. A trephine of a middle size was applied, and a circle of bone was removed extending into the cancellous structure, but no abscess was discovered. I then, by means of a chisel, removed several other small portions of bone at the bottom of the cavity made by the trephine. As I was proceeding in this part of the operation, the patient suddenly experienced a sensation which he afterwards described as being similar to that which is produced by touching the cavity of a carious tooth, but much more severe, and immediately some dark coloured pus was seen to issue slowly from the part to which the chisel had been last applied. This was absorbed by a sponge, so that the quantity of pus which escaped was not accurately measured, but it appeared to amount in all to about two drachms. From this instant the peculiar pain belonging to the disease entirely ceased, and it has never returned. The patient experienced a good deal of pain, the consequence of the operation, for the first twenty-four hours, after which there was little or no suffering. The wound was dressed lightly to the bottom with lint. Nearly six months elapsed before it was completely cicatrized: but in about three months from the day of the operation, Mr. B. was enabled to walk about and attend to his usual occupations. He has continued well to the present time (January 7, 1832); and the tibia is now reduced in size so as to be scarcely larger than that of the other leg. No exfoliation of bone has ever taken place.

"Case III. In the beginning of January 1830, Mr. S., thirty-four years of age, consulted me on account of the following symptoms.

"The lower extremity of the left tibia was considerably enlarged; the skin covering it was tense, and adhered closely to the parts below. The patient complained of a constant aching pain, which he referred to the enlarged bone. Once in two or three weeks there was an attack of pain more severe than usual, during which his sufferings were excruciating, lasting several hours, and sometimes one or two days, and rendering him altogether incapable of following his usual occupations. The pain was described as shooting and throbbing, worse during the night, and attended with such exquisite tenderness of the parts in the neighbourhood of the ankle, that the slightest touch was intolerable.

"Mr. S. said that, to the best of his recollection, the disease had begun eighteen years ago, in the following manner. On going to bed one evening he suddenly experienced a most acute pain in the inner ankle. On the following morning he was unable to put his foot to the ground, on account of the agony which every attempt to do so occasioned. Leeches were applied several times, and afterwards blisters, but the pain increased notwithstanding. After some weeks an abscess presented itself and broke. This was followed by some mitigation of the symptoms. Soon afterwards another abscess formed and broke in the neighbourhood of the first.

The two abscesses remained open for a considerable time, and then healed rapidly. Mr. S. now began to regain the use of the limb, and by degrees was able to walk as usual.

"During the following summer he had a recurrence of pain in the inner ankle, without any further formation of abscess. For eight or ten years afterwards there were occasional attacks of pain, lasting one or two days at a time; the intervals between them being of various duration, and in one instance not less than nine months. After this the attacks recurred more frequently, and during the whole of the last two years the symptoms were nearly as severe as at the time of my being consulted.

"On examining the limb, I was struck with the resemblance which it bore to that of the limb in each of the two preceding cases. There was also a remarkable resemblance in the symptoms as described by the patient, and I could not but suspect that they depended on a similar cause. I requested that Mr. Travers, who had attended one of the former cases with me, should be consulted: and he agreed with me in the opinion that probably an abscess existed in the centre of the tibia, and that it would be advisable to perforate the bone with a trephine, with the view of enabling the contents of the abscess to escape.

"Accordingly I performed the operation, with the assistance of Mr. Travers, on the 31st of January. A crucial incision was made through the skin, the angles of which were raised so as to expose a part of the bone above the inner ankle, to which the pain was especially referred. A small trephine was then applied, and a circular portion of bone was removed extending into the cancellous structure. Other portions of bone were removed with a narrow chisel. At last about a dram of pus suddenly escaped and rose into the opening made by the trephine and chisel. On further examination, a cavity was discovered from which the pus had flowed, capable of admitting the extremity of the finger. The inner surface of this cavity was exquisitely tender; the patient experiencing the most excruciating pain on the gentlest introduction of the probe into it.

"He passed a tolerable night, and suffered but little on the following day. He continued to go on favorably until the 5th of February, when a violent inflammation attacked the limb immediately above the inner ankle. In spite of the application of leeches, an abscess formed, which in the course of six or seven days presented itself immediately below the part at which the trephine had been applied. An opening was made with a lancet, and a considerable quantity of pus escaped, which had apparently formed between the periosteum and bone, the latter being felt exposed at the bottom of the abscess. During the following month the inflammation excited by the operation continued, and several abscesses presented themselves in the neighbourhood of the first. These, however, all healed favorably, without any exfoliation of bone taking place. The cavity made by the trephine became filled up by granulation, and the wound gradually cicatrized. From the time of the operation, the peculiar pain from which the patient had previously suffered was entirely relieved: and it was not long before he was quite restored to health, and able to walk and pursue his occupations without interruption. I have seen him lately, nearly two years from the time of the operation having been performed, and he continues perfectly well." (P. 239.)

Dr. Marshall Hall's "*Experimental Investigation of the Effects of the Loss of Blood*" would be injured by abridgments; we, therefore, are unwillingly compelled to pass it by unnoticed; as likewise Mr. Travers' continuation of his "*Observations on the Local Diseases termed Malignant.*"

Had we not already laid this volume under extensive contributions, Mr. Howship's memoir "*on the Phenomena and Appearances from partial Obstruction of the Cerebral Circulation*" would have furnished an interesting extract; and several might have been taken from Mr. Shaw's paper on "*a Peculiarity in the Conformation of the Skeleton in Rickets*," in which he very clearly points out the difference between rachitis, properly so called, and that morbid condition whence distortion of the spine ensues, and which is remarkable for its frequency in females, commencing usually about the age of twelve or fourteen.

The late Mr. John Shaw, it is well known, paid much attention to diseases of the osseous system; and his brother, the author of the memoir under consideration, appears to be worthily treading in his footsteps. The most important practical point arrived at by their researches is the fact that, unless there be at the same time marks of rickets in some of the long and solid bones, in whatever state of distortion the spine and ribs may be, the bones of the pelvis will not be found distorted. This is contrary to the generally received opinion; for many persons still believe that the artificial means resorted to for the support of the spine will distort the pelvis; whereas, if the principles here sought to be established are true, no such effect need be dreaded.

In order to establish this doctrine on a philosophic basis, Mr. Shaw introduces, very appropriately, a summary account of the changes which the human figure undergoes in its development; and then, from a very extended series of observations and admeasurements, shows that, in rachitis, the natural development is arrested, as well as perverted; and that the evolution of various parts, which at different ages are peculiarly active in their growth, is stayed, so that the due proportions of the several parts of the skeleton become interfered with: thus, that in rickety persons, the lower limbs remain comparatively much shorter than the upper; the head and arms of an adult being united, as it were, to the pelvis and inferior extremities of a child.

"The capacious head and diminutive pelvis of the fœtus are provisions for its safe delivery from the womb. But the same configuration, it is obvious, would be incompatible with the privilege enjoyed by man of carrying his body erect. Accordingly, before the child can leave the nurse's arms, or before the human form is completed, there is a remarkable change in the proportions of the figure: the pelvis and the legs, which form the base on which the trunk is sustained, become the larger and preponderating parts, while the head, upper extremities, and thorax are comparatively light. As an example of the extent of this alteration, the centre of gravity in the fœtus, immediately before birth, is situated as high up as the scrobiculus cordis, while in the adult, according to Borelli, it is between the two hip-joints. With the view of obtaining a more accurate conception of the change which thus take place in the relative proportions, the following measurements of the skeleton were made, in the infant newly born, and in the adult. The entire length of the child, measured for this purpose, was eighteen inches and a quarter; the dimensions of the adult were found by measuring four individuals, whose mean height was five feet eight inches. In the infant, the length from the apex of the head to the highest part of the spine of the ilium, was two inches greater than the length from the same point of the ilium to the heel; that is, the length of the superior division exceeded that of the inferior by one ninth of the entire height. In the adult, the upper division was less than the lower by sixteen inches; or, in other words, it was one fourth of the whole height shorter than the inferior division. Upon comparing the size of the head with that of the pelvis, a similar variation was observed; the circumference of the head in the newly-born child was twice that of the pelvis; whereas in the adult it was less by nearly a half. Lastly, on contrasting the length of the arms and of the legs, it was found that in the child, the former, when measured from the acromion process to the tip of the middle finger, were exactly of the same length as the latter, measured from the greater trochanter to the heel; but in the adult the arms were twelve inches shorter than the lower extremities. In illustration of the same fact, we may observe that the humerus, in the fœtus near the sixth month, is equal, both in size and weight, to the femur; but at adolescence the femur is fully three times the weight of the humerus.

"From these calculations we may perceive what a remarkable accession to its growth the inferior division of the body acquires in contrast with the superior division. We are authorized, therefore, to conclude that the lower extremities, including under that term the pelvis and the bones of the leg, advance with a greater activity between birth and manhood, than the head, thorax, spinal column, or upper extremities: and that it is owing to this difference in the rapidity of the development of the several parts of the frame that the human figure becomes changed, as we have observed, in its proportions.

"*Of the influence which Rickets has in retarding the growth and changing the proportions of the Skeleton.* If we revert now to the peculiarity of the conformation which characterizes the skeleton affected with rickets, we shall perceive that, although it differs from the adult skeleton, it bears an obvious resemblance to that of the child: the bones may be larger, they may have the firmness and strength which belong to the mature subject, the marks of the sutures and epiphyses may be obliterated; but the superior division of the frame preponderates over the lower as it does in infancy. Reflecting upon this similarity, it is naturally suggested that the cause of the remarkable form of the rickety skeleton must be a defect in the process of growth occasioned by the prevalence of that disease in the osseous system. If the interruption which we are supposing takes place while the change of the configuration just described is in the course of being accomplished, it is evident that the skeleton will retain in adolescence the peculiarities that belong to its primitive form. It might appear, at first sight, as if the peculiarity were owing to the defect of growth falling more exclusively upon the lower division of the frame than upon the upper, inasmuch as the bones of the pelvis and of the legs suffer a greater loss in their dimensions than the other parts. But this difference is to be accounted for by the process of development being more active in the lower extremities than in the superior division, as has just been shown; which makes the stoppage of the growth more strikingly exhibited in them.

"The distorted skeleton, marked No. 3 in Table 1., was found to be of the same height, when measured along the course of its curvatures, as a girl whose age was eleven years and a half; they were both fifty-one inches and a half in height. But the following contrast was observed in their relative dimensions: In the girl, the length from the apex of the head to the spine of the ilium was eleven inches and a half less than from the ilium to the heel; but in the rickety skeleton the superior division was only five inches and a half less than the inferior. The arms of the girl were nine inches shorter than the extent from the ilium to the heel; but in the rickety skeleton the difference was only three inches and a half. It thus appears, that if we desire to make a comparison between the natural skeleton and that of this deformed person, we must take the skeleton of a child still less advanced than this; that is to say, about three or four years of age." (P. 451.)

The following remarks on "the relative proportions of dwarfs and tall individuals," although not entirely new, are physiologically curious.

"The examples of dwarfs and of persons whose height surpasses the common standard, serve to illustrate our subject. They show how much the relative proportions of the body may be varied according as the growth is either retarded or accelerated. In the dwarf, where the development is a slow and imperfect process, the superior half of the body retains, at maturity, the same preponderance over the lower, which naturally belongs to it at birth. An interesting example of this is to be seen in the skeleton of the dwarf, Crachami, preserved in the Museum of the College of Surgeons.

Although this individual lived to the age of nine years and a half the skeleton exhibits the very same relative proportions which belong to the infant at the age of two or three months; indeed, if it were not for the advanced condition of the teeth, the closing together of the sutures, and the general firmness of the bones, we should not be able to distinguish it from a skeleton of that early period. In the same museum there are the portraits of five other remarkable dwarfs, among which is that of the Polish dwarf Boroawloski; and in him the peculiarity which is here described is very distinctly presented. It can at once be observed, that although his features, and the court dress with which he is equipped, bespeak a person of mature years, the size of his head, the length and capaciousness of his body, the narrowness of his hips, and the disproportionate shortness of his legs, betray the original configuration of the child. It accordingly appears that the proportions of the dwarf correspond with those of the individual affected with rickets; for in both the lower division of the body fails to acquire that superiority of bulk over the upper division which naturally belongs to the figure in adolescence.

"But as the interruption to the process of growth causes the adult to retain the peculiarities of the child's figure, so it may be shown that the acceleration of the process makes the tall individual become a true caricature of the mature form. The lower extremities being endowed with a superior activity of development, they expand and elongate in the tall person, under the increased impulse given to the whole growth, with a rapidity that soon destroys the proportions of the body. Thence it is a general remark, that persons distinguished for their unusual height, have their lower limbs disproportionately long, in comparison with the size of their trunk. To illustrate this, I took the measurements of a gentleman whose height was six feet four inches and a half, and contrasted them with the measurements of an individual of the proper proportions, whose height was five feet eight inches. It was found, in the first place, that the length from the apex of the head to the spine of the ilium was only half an inch greater in the tall person than in the other; but below the same point of the ilium, the tall individual measured as much as eight inches more than the other. 2dly, The circumference of the head was only one inch and a quarter greater in the tall individual than in the person of common proportions; but in the pelvis the difference amounted to seven inches. 3dly, The length of the arms in the tall person was less than that from the spine of the ilium to the heel by sixteen inches; while in the other individual the difference was only eleven inches and three quarters. This shortness of the arms, contrasted with the length of the legs, would have caused, it is obvious, a remarkable deformity of the figure, if the vertebral column had not also been peculiarly short: but the spine, being comparatively defective in height, allowed the arms to hang down to their usual extent in this gentleman, and thus the difference in the relative length of the upper and lower limbs was not easily noticed; yet it is to be observed, that the increase in the length of the arms was only equal to the half of what it was in the lower extremities. 4thly, The difference between the length of the upper and of the lower divisions amounted, in the tall individual, to one third of his entire height; while it was only one quarter in the person of common proportions." (P. 457.)

Without attempting to demonstrate the precise nature of that morbid condition which causes the lateral curvature of the spine, we think Mr. Shaw has shown that it does not depend upon rachitis; and, after adverting to the inconsistency of such an opinion with the phenomena observed, and the treatment most successfully pursued, he very justly continues,

"Before assuming that such formidable diseased actions gave rise to the common lateral distortion, it is remarkable that the authors have not attended more to the natural softness of texture and natural flexibility, both of the spine and the ribs, which are peculiar to the period of life when this deformity commences; and which condition is characteristic more especially of the bones of young females. As there are, however, certain circumstances unconnected with disease, which tend to increase the natural weakness of these parts, and to render them still more liable to become distorted from slight causes, I may be permitted shortly to enumerate them. 1st. The restraints to which young girls, especially in the higher classes of society, are subject, owing to the system of their education, as they interfere with the natural exercise of the body, deprive the muscles of the spine of their strength; and tend also to make the bones and the ligaments more spongy and weak. 2d. This debility of the muscular frame makes it irksome for the young patient to sit with the spine erect for any length of time; and accordingly, a disposition to lounge or to stoop is the consequence: thus the weight of the body, instead of being sustained perpendicularly upon the spinal column by the active exercise of the muscles, is allowed to hang, as it were, upon the ligaments which bind the processes of the vertebræ together, and these parts become unnaturally elongated. It is in this manner that the numerous joints of the spine, amounting to seventy-two, become relaxed, and are prepared to yield in whatever direction a particular habit may induce them. 3d. The spinal column represents a pillar of a very remarkable structure; it is not only flexible, and made to sustain a perpendicular weight, but it is supported upon a basis that is constantly shifting its level. The pelvis, on which the spine rests, is seldom for a moment placed horizontally. For example, the natural position of ease while standing, is to stand upon one leg; and accordingly the pelvis, in this posture, is necessarily elevated above its natural level on that side, and depressed below it in a corresponding degree on the other. The consequence of this inclination at the basis of the spine is that the lumbar vertebræ which are next to the pelvis, and which form together the most flexible part of the spine, are also raised unequally in one direction and fall obliquely aside on the other. Hence, in order to preserve the equilibrium, a curvature of the spine takes place in the lumbar vertebræ, while standing upon one leg. 4th. There being this curvature formed at the base, it follows that the weight of the body falls more upon the concave side of the spine than upon the convex. But it has not been observed what are all the consequences which result from this. If we attend to the natural structure of the spine, it will be seen that, whilst we lean the body to one side, the pressure is thrown, almost exclusively, upon the articulating processes of that side; these processes, delicate as they are, being the only bony structures which check the lateral movements of the trunk. Hence, when a habit is acquired of inclining to one side, or of resting upon one hip, as in sitting, the sharp edges of these small points of bone receive the weight of the entire body. But, as the articulating processes are remarkably soft, and are imperfectly formed at the age of puberty, it follows that they will become wasted by absorption when this position is long persisted in; and an inequality of the length of these two lateral props, on which the vertebræ rest posteriorly, will be the consequence, those of the concave side being shorter than those of the convex. 5th. In lateral curvature of the spine we have a distinct demonstration that the articulating processes give way more extensively than any of the other parts of the column. This is evinced by the rotation which the spine makes in its perpendicular axis, at the same time that it inclines laterally. The joints of the articulating processes being situated posteriorly as well as laterally, the spinal column cannot yield in their direction, without wheeling partially round; and it is owing to this rotation that the transverse processes, and the ribs, are directed obliquely forward upon the concave side, and obliquely backwards upon the convex side, of the curvatures, thus giving rise to a fulness or swelling on the one hand, and a depression or sinking inwards on the other. 6th. When a curvature is established in the lumbar vertebræ, it is easy to understand that a second one must follow higher up, produced by the natural efforts of the body to correct the inclination thus formed at the base of the column. With a view to preserve the equilibrium, which is disturbed, in consequence of this curve, whenever the pelvis assumes the horizontal position, one of the shoulders is elevated and the head turned aside, causing a bend to take place in the dorsal region of the spine, in an opposite direction to that situated in the lumbar. 7th. If it be admitted, that in standing, we have a natural preference to use the right leg, as being the strongest and that with which the chief efforts are made in the powerful exertions of the limbs, we may comprehend how the curvature of the lumbar region of the spine is so frequently directed with its convexity on the left side of the body, and the curvature in the dorsal region, with its convexity on the right side. The former curve results from the pelvis being depressed on its left side, as a consequence of standing upon the right leg; this inclination of the pelvis throws the spine to the left side, making it bend to the right, in order to correct the want of balance. As this curvature in the loins becomes habitual, but can only be maintained while the pelvis continues oblique, the incurvation of the dorsal region must necessarily be to the left side, or in a direction opposite to the former.

"If it be admitted from the preceding observations, that rickets has no share in producing this variety of distortion which is so frequent in females, it is obvious to what an extent the number of cases must be abridged, in which the question as to the form of the pelvis becomes a source of anxiety during pregnancy. When the accoucheur meets with a true case of deformity of the spine from rickets, he will not, it is hoped, be deceived by the notion which at present too generally prevails, that it is a mere accidental circumstance whether the pelvis in this complaint be included or not in the distortion. He will reflect, on the contrary, that if this disease has attacked the osseous system, two causes have been in operation to diminish the size of the pelvis, and, having a more correct knowledge of the different sources of the dangers in such cases, he will be prepared to meet them with greater promptitude." (P. 468.)

Dr. Lee's "*Description of the Appearances observed in a Case of Double Uterus, in which Impregnation had taken place, with Remarks on the Structure and Formation of the Membranes of the Human Ovum,*" we recommend to general perusal. We pass it by, in order to make room for Mr. Evans' "*Case in which a Cyst, containing Hydatids, was found in the substance of the Heart,*" and which, we fully agree with that gentleman, cannot "be uninteresting to the profession;" these morbid appearances being of such rare occurrence.

"The subject was an unmarried female, of about forty years of age, of slender make and weakly appearance, but who had generally enjoyed good health. During the last few years, however, she found her health somewhat decline; she was languid and indisposed to exertion, and was observed to be irritable. Since the beginning of last winter, especially, her strength failed, and she found that efforts which were formerly easy to her, were now performed with difficulty. This was particularly the case in walking up any rising ground, or mounting a staircase: such movements she avoided as much as possible in consequence, but, when she persisted in them, the result was extreme fatigue and shortness of breath.

"These symptoms increased, and, added to them, she felt occasionally a sharp pain dart through the heart; it was momentary, but most intense, so as to cause her to remark, that 'it must be with such pains that people die suddenly.' As these sufferings, however, were transient, she did not pay much attention to them, but continued her usual occupation (of nurse) until the 20th of

April, when after running down stairs and up again rather quickly, she was seized with a violent paroxysm of dyspnœa, attended by throbbing and pain of the heart, which compelled her to go to bed, and from this moment she was never again able to leave it. She remarked to her companion, that she felt as if her heart had leapt from its place. The pain subsided a little by rest, but was followed by vomiting and purging to some extent. During the night, the effort of sitting up for the purpose of relieving the bowels, induced more than once a state of complete syncope, which lasted for some time.

"On the following morning, I saw her for the first time. She was much exhausted and felt very faint; the skin was bleached, and the countenance sallow and sunken. The pulse was so rapid as to be countless, having the feel of a continued vibration of the vessel rather than of a pulse. The carotids and other large vessels also vibrated strongly. The motion of the heart was sudden, jerking, and violent; its force seemed to increase under the pressure of the hand; it was felt over a large extent of the chest, and below the sternum. The respiration was very hurried and laborious, and required a raised position of body for its performance in any degree of comfort. The pain in the chest had nearly subsided.

"By the ordinary means the diarrhœa was checked, but the sickness was rather obstinate. From this period until the time of the patient's death, there was but little variation in the symptoms. She felt constantly faint and gasping; the pulsation in the large vessels and heart became stronger, and the action of the latter was felt over a larger surface of the chest; the least exertion, even that of turning round in bed, produced great palpitation and dyspnœa; and occasionally, without any such cause, paroxysms of difficult breathing so severe as to threaten dissolution came on, and lasted for hours. Once or twice, just after such paroxysms had subsided, the pulse became for a few hours more distinct, and might almost be counted. There was little sleep, and that little was disturbed and unrefreshing. The urine was scanty, but the extremities did not swell. The legs were often affected with severe cramps. Her strength gradually failed, and on the 1st of June she died.

"The treatment was, of necessity, chiefly of the palliative kind, and but little effect was produced by it upon the disease. Once, the state of the symptoms permitting it, blood was taken from the region of the heart by leeches, and blisters afterwards applied: a slight and temporary benefit only was the result. The use of mercury combined with opium was pushed to the extent of affecting the gums: but this merely added a new source of sufferings, without relieving those already present. Large doses of opium and other anodynes had not much effect, either in alleviating the symptoms or producing sleep.

"The body was examined thirty-six hours after death. On removing the ribs, the heart had the appearance of being much larger than usual. The cavity of the pericardium contained about an ounce of fluid. The membrane was coated by a layer of coagulable

Page(s) missing

